# Evaluation of hyperbranched polyglycerol for cold perfusion and storage of donor kidneys in a pig model of kidney autotransplantation

**DOI:** 10.1002/jbm.b.34750

**Published:** 2020-10-24

**Authors:** Shadan Li, Zhongli Huang, Xiaowei Li, Youguang Zhao, Xin Jiang, Yang Wen, Hao Luo, Liang Wang, Qiunong Guan, Irina Cafeeva, Donald E. Brooks, Christopher Y. C. Nguan, Jayachandran N. Kizhakkedathu, Caigan Du

**Affiliations:** ^1^ Department of Urology The General Hospital of Western Theater Command Chengdu China; ^2^ Department of Urologic Sciences University of British Columbia Vancouver British Columbia Canada; ^3^ Department of Urology West China Hospital of Sichuan University Chengdu China; ^4^ Department of General Surgery The General Hospital of Western Theater Command Chengdu China; ^5^ Centre for Blood Research, and the Department of Pathology and Laboratory Medicine University of British Columbia Vancouver British Columbia Canada; ^6^ Department of Chemistry University of British Columbia Vancouver British Columbia Canada

**Keywords:** colloid, donor organ storage, hyperbranched polyglycerol, organ transplantation, preservation solution

## Abstract

Hyperbranched polyglycerol (HPG) is a biocompatible polyether polymer that is a potential colloid component in a preservation solution for suppressing interstitial edema during cold storage of a donor organ. This study evaluated the outcomes of kidney transplants after cold perfusion and storage with a HPG‐based preservation solution (HPGS) in a pig model of kidney autotransplantation. The left kidneys of farm pigs (weighing 35–45 kg) were perfused with and stored in either cold HPGS or standard UW solution (UWS), followed by transplantation to the right side after right nephrectomy. The survival and function of transplants were determined by the urine output, and serum creatinine (SCr) and blood urea nitrogen (BUN) of recipients. Transplant injury was examined by histological analysis. Here, we showed that there was no significant difference between HPGS and UWS in the prevention of tissue edema, but HPGS was more effective than UWS for initial blood washout of kidney perfusion and for the prevention of cold ischemia injury during cold storage. After autotransplantation, the kidneys preserved with HPGS (HPG group) had better functional recovery than those with UWS (UW group), indicated by significantly more urine output and lower levels of SCr and BUN. The survived grafts in HPG group had less tissue damage than those in UW group. In conclusion, as compared to the UWS the HPGS has less negative impact on kidney cold ischemia during cold storage, resulting in improving immediate functional recovery after transplantation, suggesting that HPG is a promising colloid for donor kidney preservation.

## 1 INTRODUCTION

In organ transplantation, a preservation solution is used for perfusion and storage of donated organs normally at cold temperature to mitigate organ injury outside the body and to improve the organ quality,^1^, ^2^, ^3^ and adding a colloid to the preservation solution is to prevent the hypothermia‐induced interstitial edema and to protect endothelial cells and other vascular cells from injury, resulting in improvement of organ functional recovery after transplantation.^4^, ^5^, ^6^ Thus, the most of the preservation solutions contain colloids as an oncotic agent,^2^ such as hydroxyethyl starch (HES) in UW solution (UWS)^3^, ^5^, ^6^ and polyethylene glycol (PEG) in IGL‐1 solution.^3^, ^7^ Among all different types of preservation solutions, HES‐containing UWS has been considered to be a standard for preservation of the liver, the kidney, and the pancreas in transplantation.^5^, ^6^, ^8^ However, several clinical trials suggest that HES causes acute kidney injury in patients with severe sepsis or undergoing artery bypass surgery^9^, ^10^, ^11^ and seriously impairs immediate donor kidney function after transplantation.^12^ In addition, HES increases the viscosity of the solution, resulting in an increase in vascular resistance of donor organs to the initial flushing/perfusion that shortens graft survival,^13^, ^14^ and it causes the hyperaggregation of red blood cells (RBCs) due to HES‐RBCs interactions,^15^ leading to incomplete blood washout from donor organs. This problem may cause poor preservation of marginal donors, evidenced by a high percentage (>50%) of delayed graft function in kidney transplants from marginal donors after preservation with UWS.^16^, ^17^ Therefore, finding a superior alternative to HES that may potentially improve the preservation of donor organs, particularly those from marginal donors, is an unmet need.

Hyperbranched polyglycerol (HPG) is a highly water‐soluble polyether polymer with approximately 50–65% dendrimeric structure.^18^ Many biological studies have reported that this polymer is blood biocompatible and does not have notable immunogenicity or toxicity in animals, and with low molecular weight (*M*_w_) it excretes preferentially via the kidneys upon intravenous injection.^18^, ^19^, ^20^ We hypothesize that the HPG may be an ideal colloidal candidate with some unique characteristics that could benefit to donor organ perfusion and storage. For instance, its low intrinsic viscosity similar to that of proteins^19^, ^20^ could result in lowering the viscosity of the cold preservation solution. As a result, it could reduce the vascular resistance of the donor organ to the initial perfusion. Indeed, replacing HES with HPG (1 kDa, 3%) in the UWS significantly reduces its relative viscosity from 3.514 to 1.378 at 4°C, more than a 2.5‐fold decrease.^21^ In rodent models, cold perfusion and storage with a HPG‐based preservation solution (HPGS) more effectively prevents the mouse organ (both hearts and kidneys) from damage than with the UWS,^21^, ^22^ and more effectively reduces kidney injury than both the UWS and HTK solution in a rat model of cold kidney ischemia–reperfusion.^23^ Unlike the rodent kidneys, the multilobular structure and intrarenal arteries and vein of the pig kidneys are similar to the human kidneys. Therefore, the pig kidneys are recommended to be a standard preclinical model for kidney transplantation research.^24^ The objective of this study was designed to evaluate the efficacy of the HPGS as compared to standard UWS in the preservation of the pig kidneys during cold perfusion and storage in a pig model of kidney autotransplantation.

## 2 METHODS

### 2.1 Polymer synthesis and characterization

HPG (1 kDa) was routinely synthesized in our laboratory by using a simple procedure of anionic ring opening multibranching polymerization as described previously.^25^, ^26^ The HPG polymer was purified by acetone precipitation and dialysis with deionized water. The chemical structure of the synthesized HPG was characterized by proton nuclear magnetic resonance (NMR) and ^13^C NMR analysis as described previously (Figure S1).^20^ By using gel permeation chromatography using a Waters 2695 Separations module (Waters Co., Milford, MA) and a DAWN HELEOS II multiangle laser light scattering detector coupled with Optilab T‐rEX refractive index detector (Wyatt Technology, Santa Barbara, CA), the average *M*_w_ (g/mol) of the HPG polymer was 820, and polydispersity index (*M*_w_/*M*_n_) was 1.34.^25^ Also the *M*_w_ (*M*_n_ = 937) was further confirmed based on the proton NMR integration data from the spectra of a Bruker Avance 300 MHz NMR spectrometer (Bruker Ltd., Milton, ON, Canada).^25^ The hydrodynamic radius of the HPG was 1.0 nm that was confirmed by using the pulsed‐field gradient NMR.^25^

### 2.2 Preparation of HPGS

The HPGS was prepared by dissolving 1 kDa HPG polymer (3%, wt/vol) in a chemical composition similar to Belzer UWS (Bridge to Life Ltd, Columbia, SC) (Table S1) as described previously.^21^, ^22^ The pH of the HPGS was measured and adjusted to 7.2–7.4 with NaOH/HCl solutions by using a Fischer Scientific Accumet pH meter (Fisher scientific, Ottawa, ON, Canada), and its osmolality (mOsm/kg) was determined by using the Advanced® Model 3,320 Micro‐Osmometer (Advanced Instruments, Inc., Norwood, MA) in the Vancouver Coastal Health Regional Laboratory Medicine (Vancouver, BC, Canada).

### 2.3 Ethics statement

All the work involving the animals in this study was performed under the protocol A11‐0409 approved by the Animal Use Subcommittee at the University of British Columbia (Vancouver, BC, Canada) according to the guidelines of the Canadian Council on Animal Care, and by the Institutional Animal Care and Use Committee and the Ethic Committee of The General Hospital of Western Theater Command (Chengdu, Sichuan, China) following the Laboratory Animal Regulations, Guidelines and Standards in China.

### 2.4 Pigs and standard UWS

Large outbred farm white pigs (female, 35–45 kg bodyweight) were purchased from the Centre for Comparative Medicine (Vancouver, BC, Canada) and were used for the kidney perfusion and storage experiments at the University of British Columbia (Vancouver, BC, Canada). Sichuan domestic pigs (35–45 kg bodyweight, 5 female and 5 male pigs in each group, *n* = 10) were from the Animal Center of Dashuo Biotechnology Co. (Chengdu, Sichuan, China), and were used for kidney transplantation at The General Hospital of Western Theater Command (Chengdu, Sichuan, China). At both sites, animals were housed in raised pens in a conventional room at 22 ± 2°C, and were fed regular pig diet with free access to tap water. Belzer UWS was purchased from the Bridge to Life Ltd.

### 2.5 Preoperative preparation, anesthesia, and cardiovascular monitoring

The preoperative preparation for all of the animals was conducted following the standardized protocol, including fasting for 12 hr with free access to water only prior to anesthesia and surgery. The premedication was ketamine (10–30 mg/kg) and midazolam (0.5–1 mg/kg) administered intramuscularly before induction of anesthesia with 3–5% of isoflurane, followed by endotracheal intubation and mechanical ventilation. General anesthesia was maintained by isoflurane (1–3%) and intravenously injection of propofol (2–6 mg/kg/hr). The arterial blood gases were kept between 75–100 mmHg pO_2_ and 35–42 mmHg pCO_2_.

### 2.6 Gravity perfusion and cold storage of pig kidneys

Following general anesthesia, the pig was placed on a warm blanket in right lateral recumbent position. An incision on the peritoneum was made via an oblique lumbar, and the left kidney was exposed through the posterior abdominal approach. The renal artery to the origin of aorta, the renal vein to the origin of vena cava, and the ureters to the level of spina iliaca were dissected. Following the administration of up to 300 IU/kg of sodium heparin, the renal artery and vein were clamped, and the donor kidney was harvested en bloc with the ureters, and immediately it was flushed with ice‐cold HPGS or UWS.

For the measures of the flow rate of organ perfusion and tissue edema, a donor kidney was perfused with the ice‐cold solution under 80 cm H_2_O pressure for 15 min. The volume of the preservation solution went through the donor kidney was recorded. After perfusion, the kidney was kept in 400 ml of the same preservation solution for 24 hr at 4°C. For the autotransplantation, immediately after harvest the left kidneys were perfused through the renal artery with either the ice‐cold HPGS or UWS (300–400 ml) under 80 cm H_2_O pressure. The perfusion was continued until the outflow at the venous end was clear as we did in clinical practice (5–8 min). After the kidneys were completely flushed, they were placed in a sterile bag containing 500 ml of the same solution (either HPGS or UWS) at 4°C for 3–9 hr.

### 2.7 Histological count of remaining RBCs after cold perfusion

A donor kidney was perfused with 400 ml of ice‐cold HPGS or UWS under 80 cm H_2_O pressure. After that, six biopsies were randomly taken from the deep cortex‐outer medulla region of a perfused kidney, followed by fixation in 10% formalin and paraffin embedding. Tissue sections were stained with hematoxylin and eosin (H&E). The RBCs were counted in randomly selected 20 glomeruli or 20 high‐powered fields (hpf, 400‐fold magnification) of corticomedullary junction of each section (one section per biopsy). The final number of RBCs per glomerulus or per hpf in the corticomedullary junction in each kidney was represented by an average number of RBCs in all of six tissue sections.

### 2.8 Determination of tissue edema

The water content in the kidney after static cold storage was used as a parameter of tissue edema. Twelve pieces of the tissue were randomly taken from each kidney after cold storage with the preservation solution. Each piece of the tissue was weighted (wet weight) prior to oven drying at 60°C. After 24 hr, the remaining tissue was weighted again (dry weight). The total content of tissue water in each piece of the tissue was assessed by measuring tissue wet weight and dry weight as follows: tissue water content (%) = (wet weight – dry weight)/wet weight. The final number of the tissue water content in each preserved kidney was represented by the average water content of the 12 pieces of the tissue.

### 2.9 Determination of tissue adenosine 5′‐triphosphate (ATP) and reduced glutathione (GSH)

The levels of both tissue ATP and GSH levels in the kidneys during the time period of cold storage was determined using ATP colorimetric/fluorometric assay (ab83355, Abcam) and GSH colorimetric assay kit (ab239727, Abcam) following the manufacturer's protocols, respectively. During the cold storage from 0 hr (immediately after cold perfusion) to 24 hr, three pieces of tissue (in triplicate) were randomly harvested from each kidney at each time point, and they were weighted (wet weight) after washing with cold phosphate buffered saline (PBS) to remove the remaining preservation solution. For the ATP determination, ATP was extracted from the tissue using phenol‐based reagents,^27^ and its level in the final extraction of each sample was quantified as compared to the ATP standards using a colorimetric microplate reader at absorbance of 570 nm (OD_570_). For the GSH measurement, the tissue was rapidly homogenized with ice‐cold 0.1 M phosphate buffer (pH 6.0) containing 5% sulfosalicylic acid and 2 mM ethylenediaminetetraacetic acid (EDTA) (1 g of tissue per 1 ml), and the supernatant was collected by centrifugation at 12,000 × g at 4°C for 20 min. After a 10‐fold dilution with GSH assay buffer, the GSH content in the supernatant was measured using the colorimetric microplate reader at OD_450_ and was calculated based on the GSH standards. The level of ATP or GSH in each kidney was presented as a mean level of the three samples.

### 2.10 Determination of lactate dehydrogenase release

Lactate dehydrogenase (LDH) release from the kidney tissue to the preservation solution was quantitated using a cytotoxicity LDH detection kit (Roche Applied Science, Laval, QC, Canada) following the manufacturers' protocols. In brief, at the each time point of cold storage from 4 to 24 hr, a small aliquot of the perfusate from the renal vein of the kidney was collected, and the level of LDH in each perfusate sample was measured in triplicate and was expressed by a mean value of OD_490_ of the three measurements of each sample.

### 2.11 Kidney autotransplantation

Kidney autotransplantation was performed using the standard technique as described previously.^28^ In brief, after static storage at 4°C, the left kidneys were transplanted orthotopically in the right kidney lodge after the right nephrectomy of the recipients. The venous anastomosis was constructed in an end‐to‐end fashion of the donor renal vein to the recipient renal vein using intermittent 6–0 prolene suture. A similar arterial end‐to‐end between the donor renal artery and the recipient renal artery using intermittent 7–0 prolene suture. Finally, the ureteral anastomosis was performed in an end‐to‐end fashion of the distal donor ureter to the recipient ureter (linked to the bladder) using continuous 3–0 vicryl suture. The time period of warm ischemia for completing anastomoses was limited to less than 30 min. Prior to completion of the arterial anastomosis, a bolus of up to 300 IU/kg of heparin was intravenously injected to prevent vascular thrombosis. Following reperfusion, furosemide (20 mg, twice a day) was intravenously administered to induce osmotic diuresis. Intraoperative antibiotics was provided by cephazolin (15 mg/kg). The kidney transplant recipients were followed up for 7 days postoperatively, until the final evaluation of blood, urine and tissue damage parameters.

### 2.12 Peri‐ and postoperative monitoring

After the operation, the permanent central venous catheter was fixed to the subcutaneous tissue of the neck of the transplant recipient pigs and were followed‐up under a controlled condition for 7 days. The vital signs were monitored 24 hr a day by animal care staff. The water and food were offered to the pig after waking up. The animals intramuscularly received duplocillin (0.05 ml/kg) every 3–5 days to prevent infection, and buprenorphine (0.01–0.05 mg/kg) every 8–12 hr as analgesics. Any urine output was collected and measured. Blood samples were taken after reperfusion every day to measure the serum creatinine (SCr) and blood urea nitrogen (BUN). When the absence of urine output was seen during a period of three consecutive days, the first day of this time period was considered the day of the graft failure.

At Day 7 posttransplantation, animals were sacrificed by the general anesthesia and consequently intravenous injection of euthanyl (120 mg/kg). The transplanted kidney was removed for histological evaluation. After completion of transplanted kidney harvest, the death was confirmed by opening the chest cavity.

### 2.13 Blood chemistry

The function of transplanted kidneys was represented by the levels of both SCr and BUN, which were measured in the Clinical Pathology Laboratory at The General Hospital of Western Theater Command by using the Dimension Vista® System with CRE2 and BUN Flex® reagent cartridges (Siemens Healthcare Diagnostics Inc., Newark, DE), respectively.

### 2.14 Histopathological assessment of tissue injury

Eight different levels of tubular damage of survived transplants at Day 7 post‐transplantation was semiquantitatively assessed by histological analysis according the scoring system as described previously.^29^ Six biopsies were randomly taken from the deep cortex‐outer medulla region of a transplanted kidney, followed by fixation in 10% formalin and paraffin embedding. The tissue sections were stained with H&E. The tubular injury in each section was semiquantitatively scored in randomly selected microscopic fields (20 views per section) in a blinded fashion. The final score of tubule injury in each graft was represented by an average score of all of six sections.

### 2.15 Statistical analysis

Data were presented as the mean ± *SD* or SEM of each group as indicated in figure legend. Statistical analysis was performed using the GraphPad Prism software (GraphPad Software, Inc., La Jolla, CA), by either two‐tailed *t*‐test or analysis of variance (ANOVA) where appropriate. The difference was statistically significant when *p* < 0.05.

## 3 RESULTS

### 3.1 Red blood cell washout from donor kidneys by perfusion with cold HPGS

**FIGURE 1:**
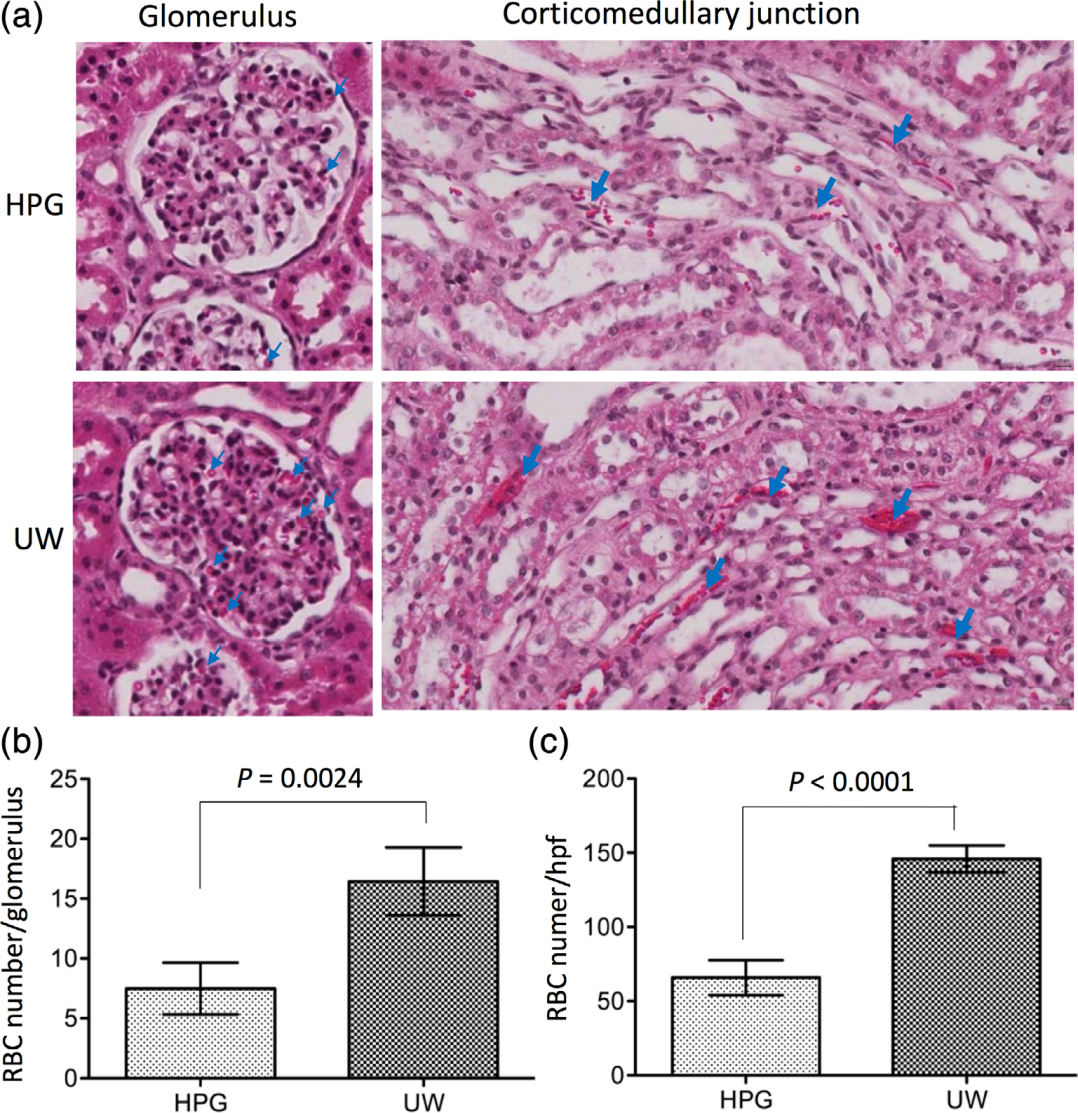
Improved red blood cell (RBC) washout from pig kidneys by cold perfusion with hyperbranched polyglycerol‐based preservation solution (HPGS) as compared with standard UW solution (UWS). Pig kidneys were perfused with 400 ml of ice‐cold HPGS (HPG group) or UWS (UW group) under 80 cm H_2_O pressure. The remaining RBCs in both glomeruli and corticomedullary junction were counted by using histological semiquantitation. (a) A typical microscopic view of a glomerulus or a high‐powered field (hpf) of corticomedullary junction of a H&E stained kidney section after cold perfusion with either the HPGS (HPG group) or the UWS (UW group). (b) RBC numbers per glomerulus. Data are presented as the mean ± *SD* of four perfused kidneys, *p* = 0.0024 (HPG vs. UW, two‐tailed *t*‐test, *n* = 4). (c) RBC numbers per hpf of the corticomedullary junction. Data are presented as the mean ± SD of four perfused kidneys, *p* < 0.0001 (HPG vs. UW, two‐tailed *t*‐test, *n* = 4)

The blood washout from a donor organ by initial perfusion with a preservation solution is required for organ procurement. Under the 80 cm H_2_O pressure, there was more of HPGS (1.6 and 1.5 L, *n* = 2) than UWS (0.8 and 0.75 L, *n* = 2) was used within 15 min of kidney perfusion, indicating that the flow rate of the HPGS was almost twofold faster than that of the UWS in pig kidney perfusion. Next, we compared the blood washout efficiency of the HPGS from the pig kidneys with that of UWS. After gravity perfusion with 400 ml of either HPGS or UWS, the remaining erythrocytes or RBCs in both glomeruli and corticomedulary junction were counted by using histological semiquantitation. As shown in Figure 1, less erythrocytes remained in the kidneys after perfusion with HPGS (HPG group) as compared to UWS control (UW group), indicated by 7.49 ± 2.17 RBCs per glomerulus in the HPG group compared to 16.46 ± 2.84 RBCs per glomerulus in the UW group (*p* = 0.0024, two‐tailed *t*‐test, *n* = 4) (Figure 1(b)), or 65.85 ± 11.78 RBCs per high‐powered field (hpf) of corticomedullary junction in the HPG group compared to 145.85 ± 9.01 RBCs per hpf of corticomedullary junction in the control UW group (*p* < 0.0001, two‐tailed *t*‐test, *n* = 4) (Figure 1(c)).

### 3.2 Effect of HPGS on the prevention of tissue edema at the end of 24‐hr cold storage

**FIGURE 2:**
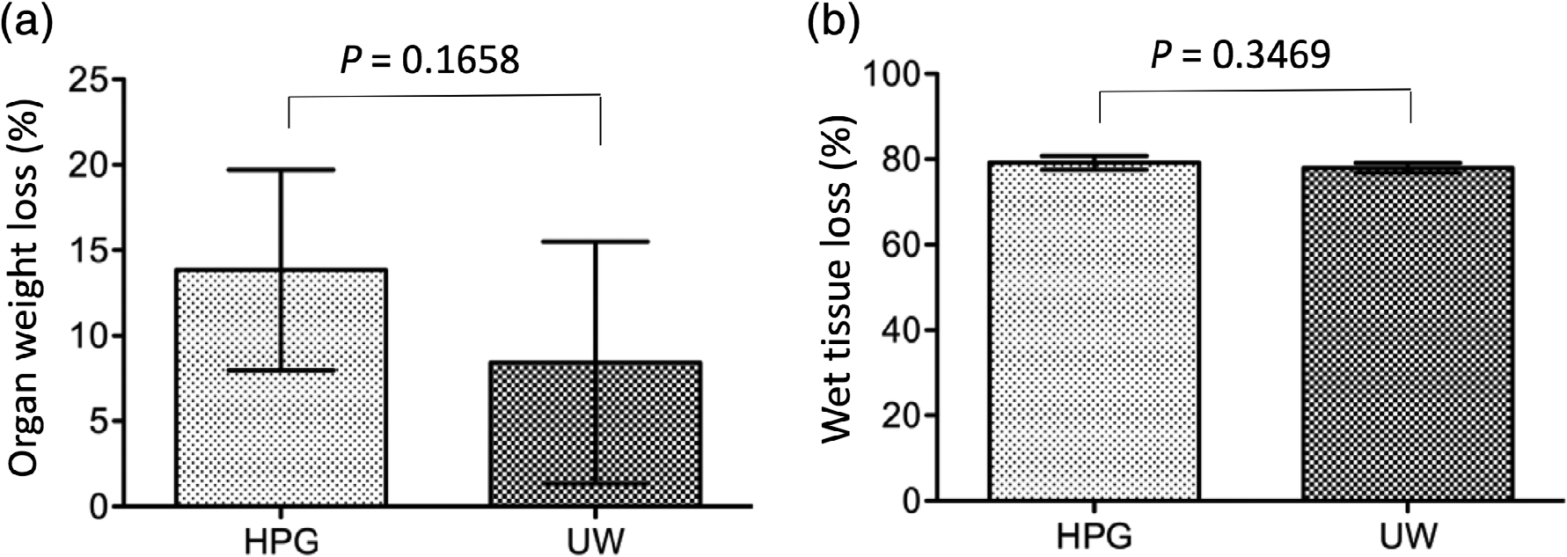
No difference between hyperbranched polyglycerol‐based preservation solution (HPGS) and UW solution (UWS) in the prevention of tissue edema during 24‐hr cold storage. Pig kidneys were perfused with either cold HPGS (HPG group) or UWS (UW group) under 80 cm H_2_O pressure for 15 min, followed by cold storage in 400 ml of the same preservation solution for 24 hr. (a) The weight change of each kidney (%) during 24 hr of cold storage. Data are presented as the mean ± *SD* in each group (HPG: *n* = 6; UW: *n* = 7), *p* = 0.1658 (HPG vs. UW, two‐tailed *t*‐test, *n* = 6–7). (b) At the end of 24 hr of cold storage, tissue water content or edema was determined by the loss of kidney tissue weight (%) after 24 hr at 60°C. Twelve pieces of tissue were randomly taken from each kidney at the end of cold storage, and the water content in each piece was measured after oven drying. Data are presented as the mean ± SD of three kidneys in each group, *p* = 0.3469 (HPG vs. UW, two‐tailed *t*‐test, *n* = 3)

Preventing cellular/tissue edema during hypothermic storage is considered the primary function of a preservation solution.^30^ The effect of HPGS was compared with that of standard UWS on the prevention of tissue edema during cold storage of pig kidneys. After gravity perfusion with either the HPGS or the UWS, the change of the kidney weight was recorded after 24 hr of cold storage in each preservation solution. As shown in Figure 2(a), the weight loss was seen in both groups, 13.85 ± 5.87% in the HPGS (*n* = 6) and 8.43 ± 7.09% in the UWS (*n* = 7), which was not significantly different (*p* = 0.1658). The degree of tissue edema at the end of 24‐hr cold storage was determined by measuring the water content in the tissue. As shown in Figure 2(b), the water content in the kidneys after cold preservation (perfusion and storage) with the HPGS was 79.19 ± 1.61%, which was not significantly different from 77.97 ± 1.16% in the kidneys with the UWS (*p* = 0.3469, n = 3).

### 3.3 Effect of HPGS on the maintenance of kidney viability during the cold storage

**FIGURE 3:**
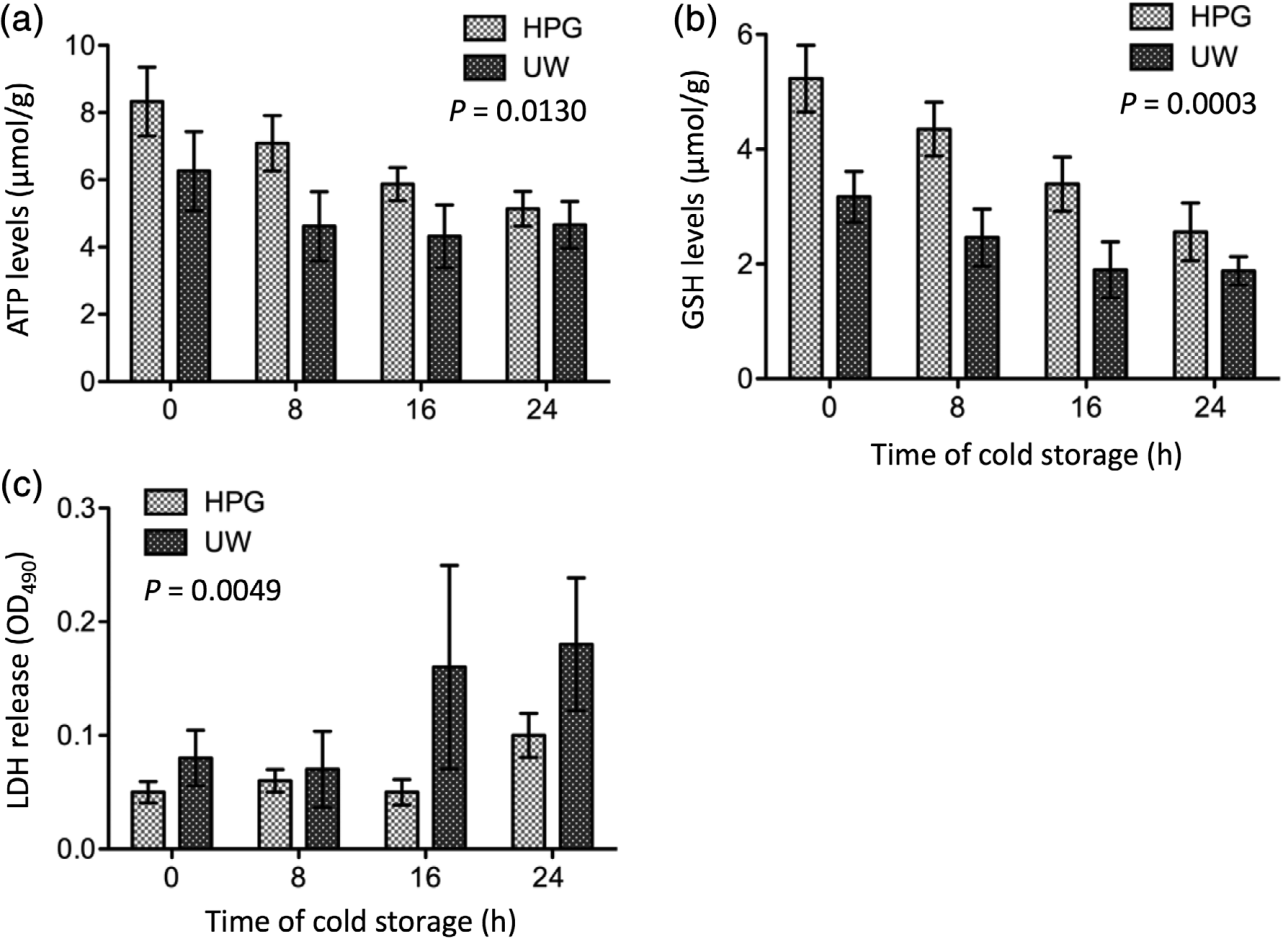
Different effects between hyperbranched polyglycerol‐based preservation solution (HPGS) and UW solution (UWS) on the maintenance of tissue viability during 24‐hr cold storage. Pig kidneys were perfused and stored with either HPGS (HPG group) or UWS (UW group) as shown in Figure 2. (a) The tissue levels of the ATP during 24 hr of cold storage. *p* = 0.0130 (HPG vs. UW, two‐way analysis of variance (ANOVA), *n* = 6–13). (b) The tissue levels of the reduced GSH during 24 hr of cold storage. *p* = 0.0003 (HPG vs. UW, two‐way ANOVA, *n* = 6–13). (c) The release of lactate dehydrogenase (LDH) from the tissue during 24 hr of cold storage. *p* = 0.0049 (HPG vs. UW, two‐way ANOVA, *n* = 4–11). Data are presented as the mean ± *SEM* of each group

The donor kidney viability is a determinant of the functional recovery after transplantation. The effect of HPGS on kidney viability during cold storage was determined directly by the tissue levels of ATP and the amount of LDH (as a cellular damage marker) release to the vascular system, and indirectly by the tissue levels of GSH, a cellular redox homeostasis marker.^31^ The ATP levels in the kidneys were decreased from 8.33 ± 1.02 μmol/g at 0 hr to 5.14 ± 0.52 μmol/g at 24 hr in the storage with the HPGS (HPG group) (*n* = 13). At the same time, the loss of ATP was significantly more with the UWS (UW group) (*n* = 6), from 6.26 ± 1.18 μmol/g at 0 hr to 4.66 ± 0.69 μmol/g at 24 hr (HPGS vs. UWS: *p* = 0.0130, two‐way ANOVA) (Figure 3(a)). The similar differences were seen in the measurement of tissue GSH between these two groups. There was a decrease from 5.23 ± 0.58 μmol/g at 0 hr to 2.56 ± 0.50 μmol/g at 24 hr in the HPG group (*n* = 13), whereas from 3.17 ± 0.45 μmol/g at 0 hr to 1.88 ± 0.25 μmol/g at 24 hr in the UW group (n = 6) (HPG vs. UW: *p* = 0.0003, two‐way ANOVA) (Figure 3(b)). Further, the LDH release from the kidney tissue to the perfusate in the vein was less (0.05 ± 0.009 at 0 hr to 0.10 ± 0.019 at 24 hr, *n* = 11) in the HPG group than those (0.08 ± 0.025 at 0 hr to 0.18 ± 0.059 at 24 hr, *n* = 4) in the UW group (*p* = 0.0049, two‐way ANOVA) (Figure 3(c)).

### 3.4 Effect of cold preservation of donor kidneys with HPGS on functional recovery after autotransplantation

Immediately after removal, left kidney was perfused with either ice‐cold HPGS or UWS until the outflow at the venous end was clear, followed by static storage in a bag containing the same solution, either ice‐cold HPGS (HPG group) or UWS (UW group). Each group consisted of 10 transplants. An ineffective blood washout was seen in all of donor kidneys after perfusion with UWS, indicated by the presence of reddish grey mark (remaining blood) in the perfusion‐discolored kidneys, whereas the discoloration was seen throughout the HPGS‐perfused kidney (Figure S2). The cold ischemia time (CIT) for each transplant was selected based on the surgeon schedule available for each operation. There was no significant difference of CIT between HPG group (3, 3, 3, 3, 6, 7.5, 7.75, 8, 8, and 9.5 hr, *n* = 10) and UW group (3, 3, 3, 3, 5, 7.25, 8, 8, 8, and 8.75 hr, *n* = 10) (*p* = 0.8806, two‐tailed *t*‐test).

**FIGURE 4:**
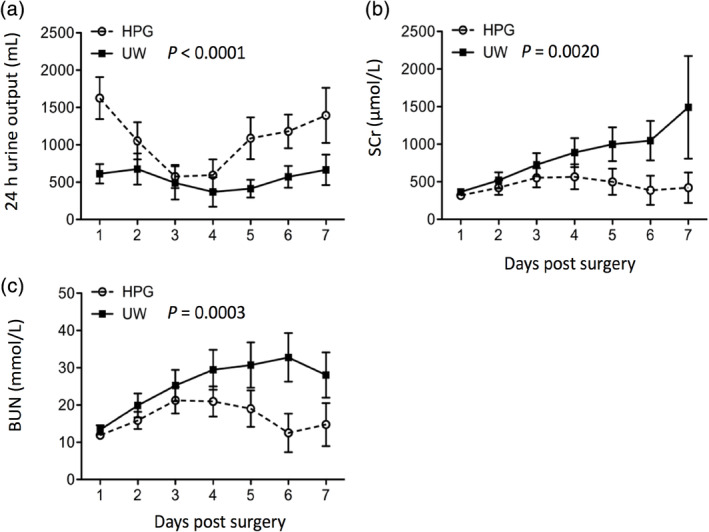
Improved functional recovery of transplanted kidneys after cold perfusion and static storage with cold hyperbranched polyglycerol‐based preservation solution (HPGS) than those with cold UW solution (UWS). The orthotopic kidney autotransplantation was performed in farm pigs under general anesthesia. The left kidneys were perfused with and stored in either HPGS (HPG group) or UWS (UW group), followed by autologous transplantation to the right side after right nephrectomy. (a) 24‐hr urine output from each transplant recipient. *p* < 0.0001 (two‐way analysis of variance [ANOVA]). (b) Serum creatinine (SCr) levels of transplant recipients in each group. *p* = 0.0020 (two‐way ANOVA). (c) Blood urea nitrogen (BUN) levels of transplant recipients in each group. *p* = 0.0003 (two‐way ANOVA). Data are presented as the mean ± *SEM* of each group (*n* = 10)

After transplantation, there were two failed grafts, indicated by the absence of urine excretion for consecutive 3 days, in each group due to severe blood clot or thrombosis. The function of grafts was primarily determined by three parameters: The amount of urine excretion from transplanted kidneys, and levels of both SCr and BUN in recipient pigs. As shown in Figure 4(a), the 24‐hr urine output in the HPG group was declined from 1626 ± 280.89 ml at Day 1 to 576 ± 154.20 ml at Day 3, and then it increased to 1395 ± 368.67 ml at Day 7. Whereas in the UW group, it started with 612.5 ± 193.18 ml at Day 1 to 490.5 ± 223.59 ml at Day 3, and ended with 666 ± 203.72 ml at Day 7 (HPG vs. UW: *p* < 0.0001, two‐way ANOVA). The increase of SCr was seen in both groups, but in the HPG group the increase was significantly less (317.57 ± 42.04 μmol/L at Day 1 to 421.12 ± 203.79 μmol/L at Day 7) than that in the UW group (366.01 ± 47.92 μmol/L at Day 1 to 1491.21 ± 683.04 μmol/L at Day 7) (*p* = 0.0020, two‐way ANOVA) (Figure 4(b)). Similarly, the lower BUN levels were seen in the HPG group as compared to the UW group (HPG: 11.91 ± 0.838 mmol/L at Day 1 to 21.29 ± 3.564 mmol/L at Day 3, then decreased to 14.76 ± 5.762; UW: 13.34 ± 1.243 mmol/L at Day 1 to 28.08 ± 6.081 mmol/L at Day 7) (*p* = 0.0003, two‐way ANOVA) (Figure 4(c)). These data suggest that HPGS‐preserved kidneys might have better functional recovery as compared to UWS‐preserved kidneys after transplantation back to the animals.

### 3.5 The tissue damage in the kidney transplants after cold preservation with HPGS

At the experimental endpoint (Day 7 post surgery), eight survived grafts from each group were collected for histological analysis of the tissue damage. In the HPG group, the color of all eight grafts was pink, and there was no noticeable blood clot inside (Figure S3). Whereas in the UW group, the color and gross structure of two grafts were not different from those in the HPG group, but the rest of six grafts were brownish pink with dark color marks on the surface, and had blood clot mostly in the area of the segmental arteries (Figure S3).

**FIGURE 5:**
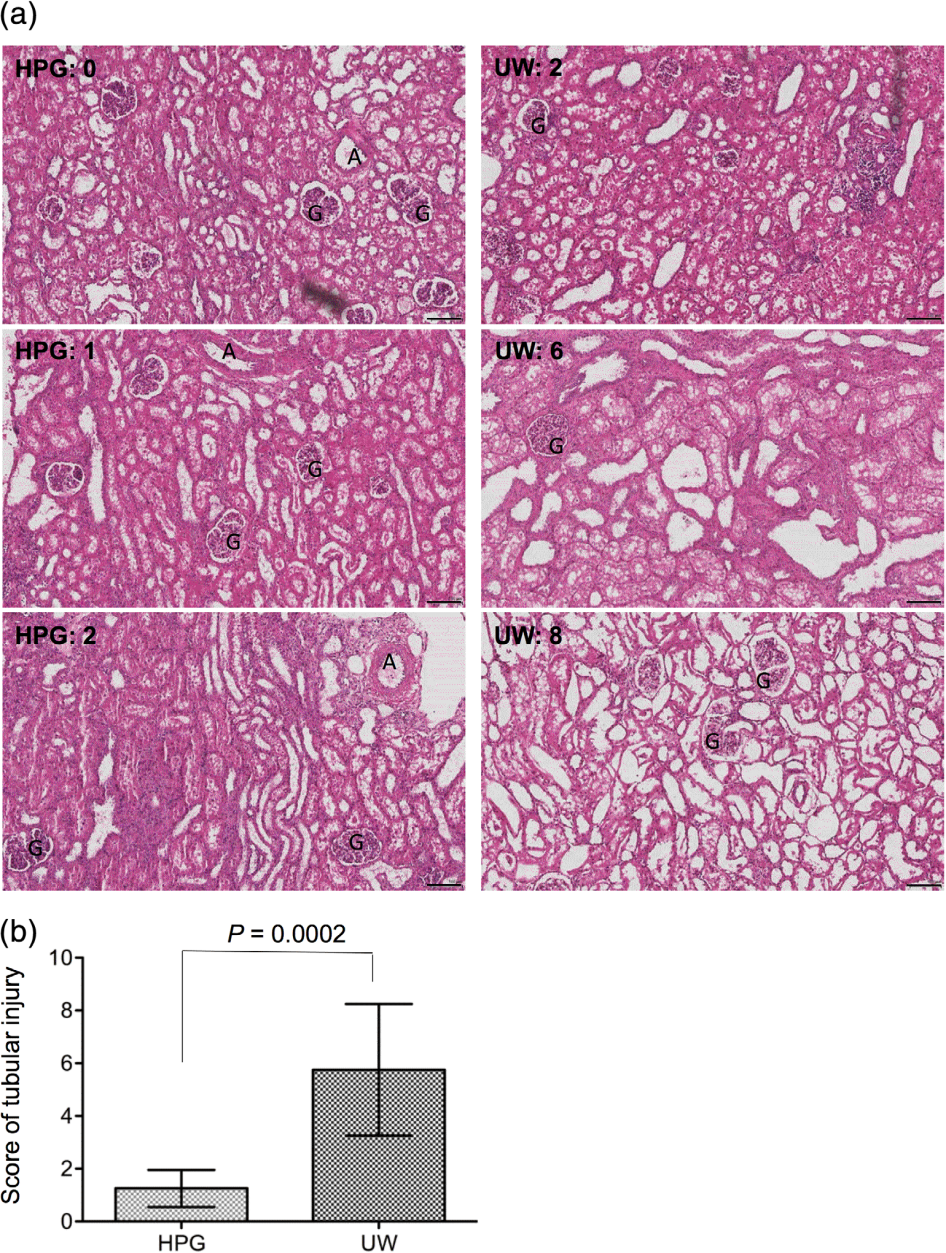
Tissue damage of transplanted kidneys at Day 7 post‐transplantation. The tissue damage of each survived transplant was examined by histological scoring of tubular injury of randomly selected six tissue sections (H&E stain). (a) Typical microscopic views showing different scores of tubular injury in each group. Left column: score 0–2 in the hyperbranched polyglycerol (HPG) group; right column: score 2–8 in the UW group. “G” glomerulus, “A” artery. Bar: 100 μm. (b) Tubular injury scores of each graft in each group. The level of tubular injury in each graft is presented as an average score of six sections, and data in each group are presented as the mean ± *SD* of eight grafts, *p* = 0.0002 (two‐tailed *t*‐test, *n* = 8)

The survived grafts of the HPG group showed normal structure to mild tubular injury (score 1–2) that was mostly characterized by acute tubular necrosis and some cellular infiltrates in the perivascular areas (Figure 5(a)). Whereas in the UW group the severe tubular injury was mainly presented by flattening, vacuolization and denudation of renal tubules (from score 2–8)(Figure 5(a)). Statistical analysis of histological scores showed that the tubular damage in the cortex in the HPG groups (score: 1.25 ± 0.707) was significantly less severe than that in the UW group (score: 5.75 ± 2.493) (*p* = 0.0002, two‐tailed *t*‐test, *n* = 8) (Figure 5(b)).

## 4 DISCUSSION

HPG is a synthetic globular‐like hyperbranched polymer,^18^ whereas HES is a modified starch, a mixture of amylose with a linear structure and amylopectin with a branched structure. Inclusion of HES in the UWS or others results in high viscosity and induction of RBC hyperaggregation,^15^ which is a disadvantage factor for its use in organ perfusion. Unlike HES, HPG did not induce RBC hyperaggregation, and it was more effective in the blood washout and prevented more tissue damage than HES in the cold perfusion of mouse kidneys.^22^ Furthermore, in a rat model of cold ischemia–reperfusion injury, less reperfusion injury was seen in the kidneys after cold perfusion/storage with the HPGS than those with HES‐based UWS or HTK solution.^23^ The present study using the pig kidneys confirms the data from these previous studies. RBCs in the pig kidneys are more effectively washed out by perfusion with the HPGS than those with the UWS (Figure 1) and less cellular damage or LDH release during cold storage with the HPGS as compared to the UWS (Figure 3(c)). These benefit effects of the HPGS are associated with better functional recovery (Figure 4) and less tissue damage (Figure 5) after transplantation.

The function of HES in the UWS is to prevent interstitial edema during cold perfusion and storage of donor organs.^5^, ^6^ Our data show that under the same colloid osmotic pressure, HPG (1 kDa, 3%) equally suppresses the tissue edema in rat kidneys^23^ as well as in pig kidneys (Figure 2) after intravascular flush and static storage. Physicochemical analysis shows that 1 kDa HPG is a compact, small polymer with 1 nm of hydrodynamic radius, and it has as an osmotic reflection coefficient (*σ*) approximately 0.175.^25^ Our data suggest that HPG (1 kDa) may act as both an oncotic agent and an impermeant in the preservation solution, which could prevent water from the organ preservation solution in the intravascular space moving into the interstitial and intracellular compartments as effectively as HES does during the perfusion and storage at cold temperature.

The high viscosity of the UWS causes an initial poor perfusion of the grafts,^13^, ^32^ and an incomplete distribution of the UWS was noted in the donor organ.^33^ Replacing the HES with the HPG (1 kDa, 3% wt/vol) in the UWS results in a 2.5‐fold decrease in its relative viscosity,^21^ resulting in increasing perfusion rate and more blood washout in the pig kidneys in this study (Figure 1). Similar observation is seen in the mouse kidneys.^22^ The functional recovery of the kidneys after cold perfusion and static storage with the HPGS is significantly improved as compared with those with the UWS in the pig model (Figure 4). The data are similar to that of rat studies as reported previously.^23^

The mechanisms underlying the relatively less harmful effects of using HPG instead of HES polymer for organ preservation (cold perfusion and static storage) are not completely understood. It is well known that HES induces hyperaggregation of RBCs^15^, ^22^, ^34^, ^35^ and induces RBC agglutination in a rat liver washout model.^36^ HES is recognized as the only component in the UWS responsible for increasing branched rouleaux formation of RBCs.^13^, ^15^ Whereas HPG inhibits rouleaux formation of RBCs.^22^ This difference may explain why the initial perfusion with the HPGS results in more complete blood washout as compared with the HES‐based UWS. Consequently, the presence of RBC rouleaux or hyperaggregation in the UWS‐perfused kidneys may prevent further adequate microvascular perfusion with the solution during procurement, resulting in occlusion of the microvasculature and subsequently causes local warm ischemia “micro”‐injury of grafts during blood reperfusion or after transplantation. Furthermore, we have previously shown that HPG is superior to HES in the maintenance of cell membrane fluidity and intracellular ATP of cultured cells at cold temperature.^21^ Similar to that, the higher levels of the ATP and GSH in the HPGS‐stored kidneys are found as compared to UWS controls during cold storage (Figure 3(a–b)). These data may suggest that HPG has advantage over HES for an increase in cell survival during cold preservation, which is supported by less LDH release from the cultured cells^21^ or from the whole pig kidney (Figure 3(c)).

## 5 CONCLUSIONS

We have demonstrated that the HPGS preserves the kidney organs better than the UWS in a pig model of kidney autotransplantation. Our data may suggest that replacing the HES with small, compact HPG in the UWS abrogates the adverse effects of the HES on slowing perfusion rate, and causing RBC agglutination during cold perfusion of donor kidneys and tissue injury during cold storage. As a result, HPGS significantly improves the functional recovery of transplanted kidneys after transplantation as compared to UWS. Taken together, our preclinical studies indicate that the HPGS could have the potential to improve the outcomes of current standard organ preservation. However, further clinical studies are warranted to confirm these findings.

## 6 LIMITATION OF THE STUDY

There are some model‐related limitations of this study. First, this is an experimental study using a limited number of farm pigs, of which the demography is not comparable to the transplant population. Second, large pig kidneys are similar to humans in size and anatomical structure, and are considered to be an ideal model for the evaluation of the organ preservation solutions in kidney preservation,^24^ but they have different genomes. Thus, the physiopathological responses, such as endothelial and epithelial cell signaling to cold temperature and interactions with the HPG polymer or HES, of between the pig kidneys and human counterparts may not be the same. Third, the blood clotting profiles between pigs and humans are different. For example, the clotting time (146–296 s) or the clot formation time (30–84 s) are found in the pig blood without thrombin, which are threefold faster than those (the clotting time: 476–901 s; the clot formation time: 104–436 s) of the human blood, and the maximum clot firmness of the pig blood is larger than that of the human blood (pig: 68–79 mm; human: 49–65 mm).^37^ All these suggest that the initial blood washout from the pig kidneys by the perfusion with a preservation solution (i.e., UWS vs. HPGS) may not be as effective as that from the human kidneys, indicating that the blood washout from the pig kidneys is of less relevance for this clinical situation.

## CONFLICT OF INTEREST

Donald E. Brooks, Christopher Y. C. Nguan, Jayachandran N. Kizhakkedathu, and Caigan Du are inventors of “Polymer based transplant preservation solution” (WO2015074139A1). The other authors declare no conflicts of interest.

## Supporting information


**Figure S1** Chemical structure of HPG polymer.

**Figure S2** Photograph of a typical donor kidney after perfusion and static storage with ice‐cold HPGS or UWS. Star: reddish grey mark.

**Figure S3** Photographs of a typical kidney transplant at day 7 post‐transplantation. HPG group (upper panel): left image; a typical transplant before harvest, right image; the transplant cross‐section. UW group (bottom panel): left image; a transplant before harvest, right image; the transplant cross‐section. Arrows: blood clot.

**Table S1** Composition of organ preservation solutions.
