# Supplementary figures and images for: Evaluation of hyperbranched polyglycerol for cold perfusion and storage of donor kidneys in a pig model of kidney autotransplantation
Source: J Biomed Mater Res B Appl Biomater. 2020 Oct 24;109(6):853–63. doi: 10.1002/jbm.b.34750 (PMC8246781; doi:10.1002/jbm.b.34750)

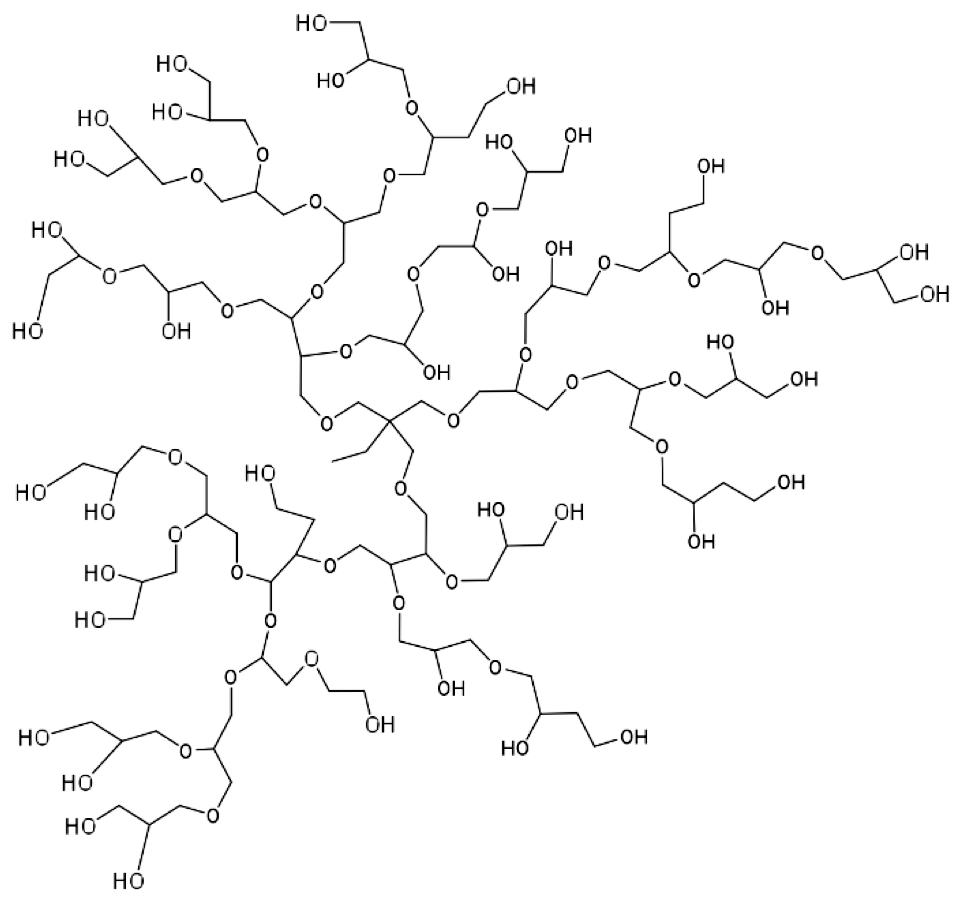

Supplement: Supplementary file 1 — Figure S1 Chemical structure of HPG polymer. [file JBM-109-853-s001.tif]

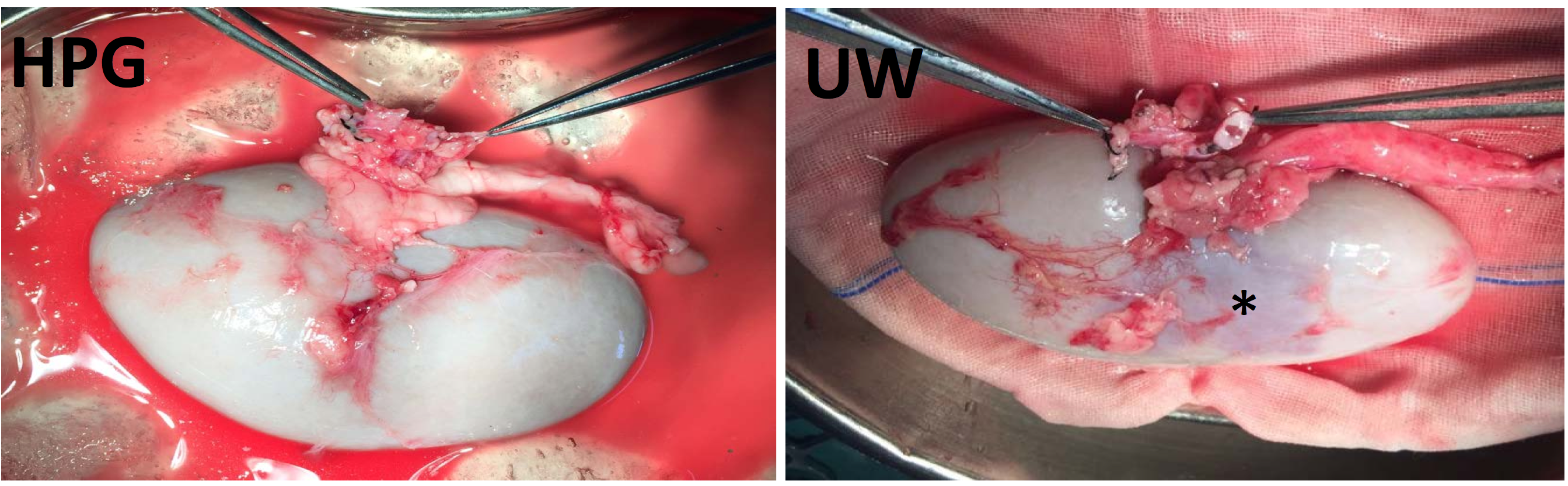

Supplement: Supplementary file 2 — Figure S2 Photograph of a typical donor kidney after perfusion and static storage with ice‐cold HPGS or UWS. Star: reddish grey mark. [file JBM-109-853-s002.tif]

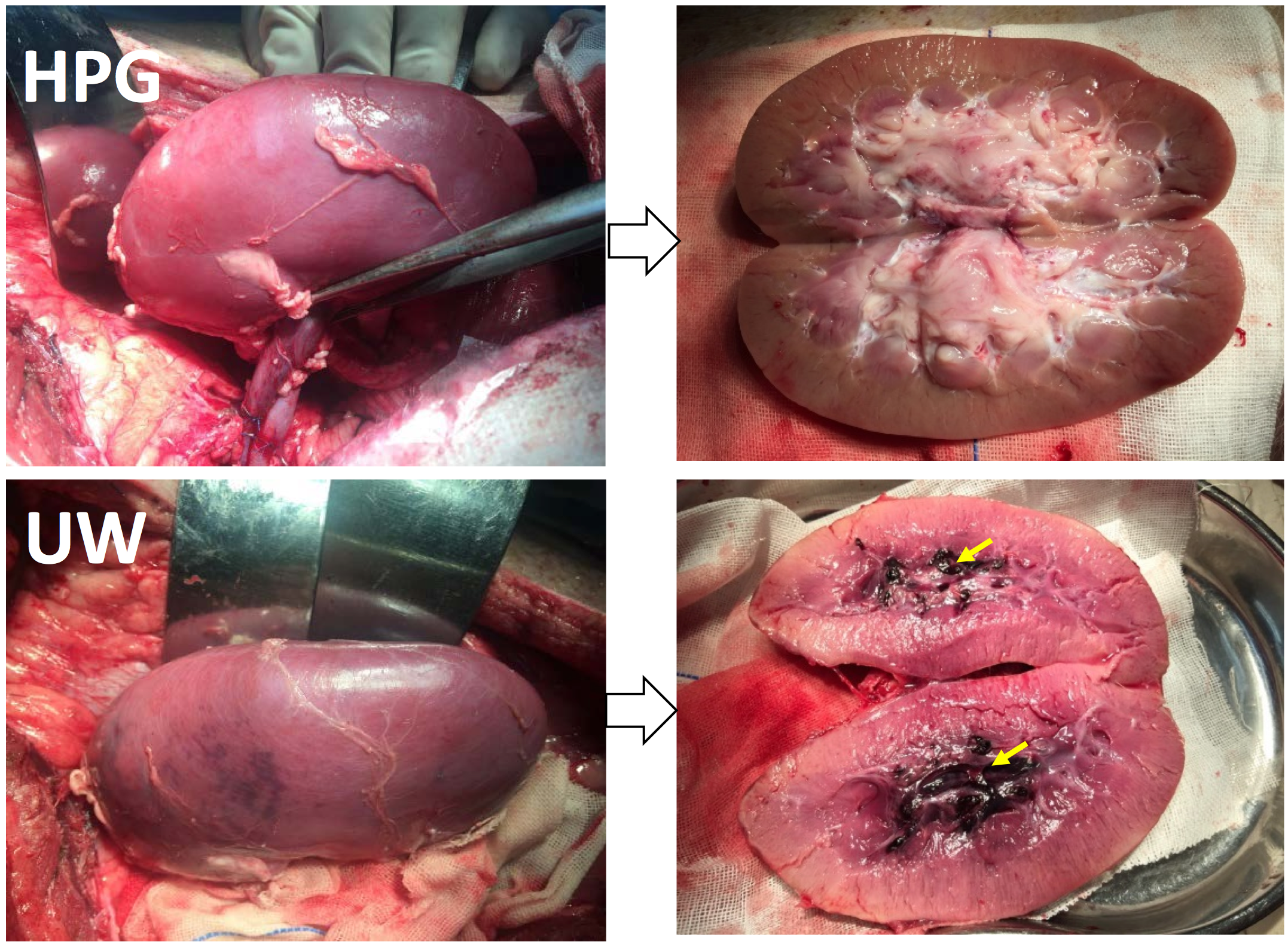

Supplement: Supplementary file 3 — Figure S3 Photographs of a typical kidney transplant at day 7 post‐transplantation. HPG group (upper panel): left image; a typical transplant before harvest, right image; the transplant cross‐section. UW group (bottom panel): left image; a transplant before harvest, right image; the transplant cross‐section. Arrows: blood clot. [file JBM-109-853-s003.tif]
