# Supplementary material for: Evaluation of hyperbranched polyglycerol for cold perfusion and storage of donor kidneys in a pig model of kidney autotransplantation
Source: J Biomed Mater Res B Appl Biomater. 2020 Oct 24;109(6):853–63. doi: 10.1002/jbm.b.34750 (PMC8246781; doi:10.1002/jbm.b.34750)
Supplement: Supplementary file 4 — Table S1 Composition of organ preservation solutions. [file JBM-109-853-s004.docx]

**TABLE S1** Composition of organ preservation solutions

| Chemicals | UW solution | HPG solution |
| --- | --- | --- |
| 100 mM Lactobionic acid | + | + |
| 100 mM KOH | + | + |
| 25 mM KH_2_PO_4_ | + | + |
| 5 mM MgSO_4_ | + | + |
| 5 mM Adenosine | + | + |
| 3 mM Glutathione | + | + |
| 1 mM Allopurinol | + | + |
| 30 mM Raffinose | + | ˗ |
| 50 g/L Hydroxyethyl starch | + | ˗ |
| 30 g/L HPG | ˗ | + |
| pH 7.4 | + | + |
| Osmolarity: ~320 mOsm/kg | + | + |

UW solution was purchased from Bridge to Life (USA). HPG solution was prepared in Dr. Du’s lab by dissolving HPG (~ 1 kDa, 3%, w/v) in a solution containing the same composition as in UW solution but omitting 30 mM raffinose (an unnecessary component) and 5% hydroxyethyl starch (replaced by 3% HPG). The pH of HPG solution is adjusted to 7.2-7.4 by using NaOH/HCl at 22 °C.
